# Respiratory Symptoms of Vendors in an Open-Air Hawker Center in Brunei Darussalam

**DOI:** 10.3389/fpubh.2014.00167

**Published:** 2014-10-02

**Authors:** Nurul Nor Nazurah bt Abdul Wahid, N. B. P Balalla, David Koh

**Affiliations:** ^1^PAPRSB Institute of Health Sciences, Universiti Brunei Darussalam, Bandar Seri Begawan, Brunei Darussalam; ^2^Occupational Health Division, Ministry of Health, Bandar Seri Begawan, Brunei Darussalam; ^3^Saw Swee Hock School of Public Health, National University of Singapore, Singapore

**Keywords:** cooking vendors, respiratory symptoms, charcoal, LPG, open-air cooking

## Abstract

**Objectives:** We studied respiratory problems among vendors exposed to cooking fumes in an open-air hawker center. Exposure to cooking fumes from either the use of fossil fuels or liquefied petroleum gas (LPG) has been shown to be associated with adverse respiratory health effects.

**Methods:** We conducted a cross-sectional study among 67 food vendors exposed to cooking fumes as well as 18 merchandise sellers at an open-air hawker center in Brunei Darussalam. Past medical and smoking history and exposure to cooking fumes were obtained. The validated American Thoracic Society Questionnaire with a translated Malay version was used to ask for respiratory symptoms.

**Results:** Compared to merchandise sellers (*n* = 18), cooking vendors (*n* = 67) had a higher self-reported respiratory symptoms (50.7% for those cooking and 33.3% for merchandise sellers). Cough (28.3%) was the main respiratory symptom experienced in cooking vendors and breathlessness (22.2%) among merchandise sellers. Half (50.0%) of cooking vendors who worked for more than 10 years had cough and 27.3% had phlegm. Those cooking with charcoal were two times more likely to have cough than those cooking with LPG. Cooking vendors with a job duration of more than 10 years were thrice more likely to have cough.

**Conclusion:** Cooking vendors in the open-air hawker center exposed to cooking fumes had more respiratory symptoms compared to non-exposed merchandise sellers. The type of fuel used for cooking and duration of work was associated with increased prevalence of cough.

## Introduction

Numerous studies have indicated that cooking fumes, either fossil fuels or liquefied petroleum gas (LPG), contain a considerable level of polycyclic aromatic hydrocarbons (PAHS), oxides of nitrogen (NOx), carbon monoxide (CO), and other compounds, which can cause undesirable health consequences. Vainiotaloa and Matveinena (1) confirmed that cooking fumes contain hazardous compounds such as formaldehyde, acetaldehyde, acrolein, fat aerosol, and PAHS compounds. They stated that workers cooking with these fuels may be exposed to relatively high concentrations of airborne impurities.

According to a paper by Johnson (2), charcoal grilling, which has a higher heating value, has a footprint of 998 kg CO_2_e, which is almost three times that for grilling with LPG, 348 kg CO_2_e. Grilling with LPG has a fuel consumption that is relatively close to the amount of food cooked as the fuel consumption using LPG can be easily regulated compared with charcoal.

There are several health effects associated with exposure to cooking fumes, which have been reported include not only increased risk for respiratory symptoms but also of non-respiratory outcomes such as cancer and adverse reproductive outcomes.

In a review by Basu and Samet (3), their conclusion was that nitrogen dioxide (NO_2_) emitted by gas stoves is not risk-free. The indoor use of gas stoves was linked with lower respiratory symptoms (OR = 1.23; 95% CI = 1.03, 1.47) and with chronic respiratory conditions (OR = 2.08; 95% CI = 1.49, 2.90). Women, in particular, were at increased risks of wheezing (OR = 2.07; 95% CI = 1.41, 3.05), waking with shortness of breath (OR = 2.32; 95% CI = 1.25, 4.34), and having asthma attacks (OR = 2.60; 95% CI = 1.20, 5.65). A study (4) in Singapore showed that acute short-term exposure to NO_2_ during cooking was significantly correlated with the fall in peak expiratory flow rate (PEFR) of 3.4% (*r* = 0.579; *P* = 0.019).

For non-respiratory effects, See et al. (5) showed that cooking fumes in three commercial kitchens posed a higher carcinogenic risk to exposed workers. The excess lifetime cancer risk (ELCR) of employees were beyond the recommended acceptable ELCR limit of 10^−6^ in all three kitchens studied (4.08 × 10^−3^, 1.21 × 10^−2^, 1.07 × 10^−3^).

Another study (6) showed women who cook with biofuels are twice as likely to have experienced two or more stillbirths as those who cook with cleaner fuels (RR = 2.01; 95% CI: 1.11, 3.62).

We studied respiratory health problems in vendors at the Gadong night market; a major open-air center for vendors in Brunei Darussalam. The specific objectives were to compare the prevalence of these symptoms in food vendors and merchandise sellers and to identify any association between age, gender, and social factors (i.e., smoking, pets, and hobbies), job duration, and type of cooking fuels used with respective respiratory symptoms. This study was done in an open-air market as our literature review showed that previous studies (7–9) were mostly in indoor environments.

## Materials and Methods

A total of 120 survey forms were distributed to all the vendors in the market; where only the two officially registered respondents per stall were selected to receive the forms. Respondents were approached individually with a “Participant Information Sheet” and consent obtained to participate in the study. The respondents had to be aged 18 years or above and free from pre-existing medical conditions prior to starting work.

**Figure 1:**
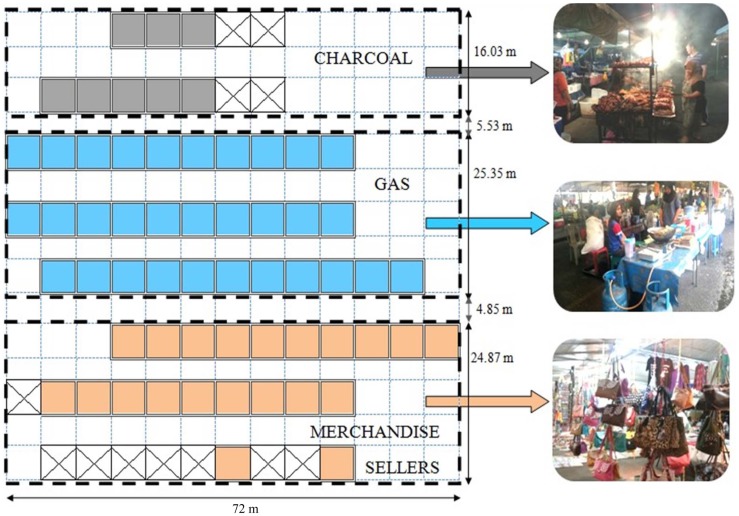
**Map of the study area, with pictures of the vendors using charcoal, gas or selling merchandise**.

Merchandise sellers nearby were used as the comparison group; the study area were separated into respective sections namely one area for those cooking with charcoal, one for those cooking with LPG, and another for merchandise sellers. Figure 1 is a map of the study area.

A modified version of the American Thoracic Society (ATS) questionnaire was used. Personal details such as name and contact number in addition to supplementary questions for adults and the children’s questionnaire were removed. A Malay version of the questionnaire was also produced, which was translated and then back-translated; ultimately all the questionnaires were self-administered and filled in Malay.

For the purpose of this study, respiratory problem is defined as current presence of at least one or more respiratory symptoms, i.e., cough, phlegm, wheeze, breathlessness, and chest cold and chest illnesses. Definitions of individual symptoms are as follows:

- Cough – a sudden audible expulsion of air from the lungs, with first cigarette smoked or on first going outdoors.
- Phlegm – thick mucus secreted by tissues lining the airways of the lungs, with first cigarette smoked or on first going outdoors.
- Wheeze – a form of rhonchus, characterized by high or low-pitched sound as a result of narrowing of the airways occurring occasionally or frequently.
- Breathlessness – a distressful sensation of uncomfortable breathing, with or without physical exertion.
- Chest cold and chest illnesses – colds in the head or chest illnesses accompanied by cough and sputum more than half the time in the past 3 years.

Exposure to charcoal and LPG fumes evaluated in this study is classified as long (>10 years), intermediate (6–10 years), or short (5 years or less) job duration. Current exposure in terms of days per week were characterized as short (1–2 days per week), moderate (3–5 days per week), and long (>5 days per week). Daily exposure to fumes were classified as low (1–2 h per day), moderate (3–5 h per day), or long (>5 h per day).

Smoking history was assessed in terms of pack years after obtaining number of cigarettes smoked per day and age when started and/or stopped for those who previously smoked. Specific questions on pets (i.e., cats, rabbits, birds) and hobbies that may expose respondents to other fumes and dust (e.g., paint work) were included.

We obtained approval to conduct this study from the Ministry of Health Research and Ethics Committee (MHREC) and Institute of Health Science Research and Ethics committee (IHSREC), and permission from both the Ministry of Health and the Municipal Board.

### Statistical analysis

The SPSS Version 20.0 was used for analysis. Fisher’s exact tests were used to state respiratory symptoms among the cooking vendors and merchandise sellers and also in identifying the presence of any association between short and long-term exposure of cooking fumes with respiratory symptoms. Simple and multiple logistic regression analysis was used to determine if there was any association between respiratory problems experienced and age, gender, exposure to cooking fumes, and social factors. The overall model fitness of multiple logistic regression was checked by Hosmer–Lemeshow goodness-of-fit test and also by percentage of cases correctly classified (>65% was considered good fitness).

## Results

### General characteristics of vendors

Table 1 summarizes the general characteristics of the participants in the study. Eighty-five vendors participated in the survey, of whom 67 were exposed to cooking fumes and 18 were non-cooking vendors. Fifty-one cooked with LPG while 16 cooked with charcoal.

**Table 1:** **Characteristics of vendors**.

Characteristics	Cooking vendors (*n* = 67)	Non-cooking vendors (*n* = 18)
	
	Frequency (%)	
Gender
Male	38 (56.7)	7 (38.9)
Female	29 (43.3)	11 (61.1)
Age (years)
18–24	30 (44.8)	7 (38.9)
25–34	19 (28.4)	5 (27.8)
35–44	11 (16.4)	4 (22.2)
45–55	4 (5.9)	1 (5.5)
>55	3 (4.5)	1 (5.5)
Ethnicity
Malay	64 (95.5)	17 (94.5)
Others	3 (4.5)	1 (5.5)
Job duration (years)
0–5	32 (47.8)	9 (50.0)
6–10	13 (19.4)	4 (22.2)
11–15	22 (32.8)	5 (27.8)
Duration of working days
1–2	–	–
3–5	10 (14.9)	6 (33.3)
>5	57 (85.1)	12 (66.6)
Duration of working hours
1–2	2 (3.0)	0 (0.0)
3–5	13 (19.4)	2 (11.0)
>5	52 (77.6)	16 (89.0)
Smoking
Yes	29 (43.3)	5 (27.8)
No	38 (56.7)	13 (72.2)
Fuel used at sites
Liquefied petroleum gas	51 (76.1)	–
Charcoal	16 (23.9)	–

In total, there were 35 non-respondents, 30 of whom cooked with LPG and 5 of whom were merchandise sellers.

The food was cooked using one or more of the following methods: deep-frying, stir-frying, simmering, steaming, and grilling (with charcoal). Non-cooking vendors were selling a variety of merchandise such as clothes, toys, accessories, fresh fruits, and vegetables. In general, cooking vendors have been in their profession and have occupied the study area longer than the merchandise sellers (Table 1).

### Prevalence of respiratory problems

The overall prevalence of any respiratory symptom was 47.1%. Cough (24.7%) was the leading respiratory symptom among all respondents. It was also the key respiratory symptom experienced by cooking vendors (28.4%). Those using charcoal (43.8%) had higher prevalence compared to those using LPG (23.5%). Breathlessness (22.2%) was the main respiratory symptom experienced among the non-cooking vendors (Table 2).

**Table 2:** **Prevalence of respiratory symptoms and problem in vendors**.

Variable	*n*	Cough	Phlegm	Wheeze	Breathlessness	Cold and chest illnesses (%)	Any respiratory problem
	
		Frequency (%)					
**COOKING FUEL**							
Charcoal	16	7 (43.8)	0 (0.0)	1 (6.3)	1 (6.3)	0 (0.0)	8 (50.0)
LPG	51	12 (23.5)	9 (17.6)	8 (15.7)	12 (23.5)	12 (23.5)	26 (51.0)
Cooking	67	19 (28.4)	9 (13.4)	9 (13.4)	13 (19.4)	12 (17.9)	34 (50.7)
Non-cooking	18	2 (11.1)	3 (16.7)	3 (16.7)	4 (22.2)	0 (0.0)	6 (33.3)
All	85	21 (24.7)	12 (14.1)	12 (14.1)	17 (20.0)	12 (14.1)	40 (47.1)

There was an increasing trend of cough prevalence in merchandise sellers (11.1%), vendors cooking with LPG (23.5%), and charcoal (43.8%) (*P* = 0.104) (Table 3).

**Table 3:** **Relationship between cough and different exposures**.

Variable	*n*	Cough	No cough	*P* value^a^
	
		Frequency (%)	
Non-cooking vendor	18	2 (11.1)	16 (88.9)	0.217^a^
Cook with LPG	51	12 (23.5)	39 (76.5)	
Cook with charcoal	16	7 (43.8)	9 (56.3)	

*^a^Fisher’s exact test (comparing between cooking and non-cooking groups)*.

### Association between job duration and respiratory symptoms among cooking vendors

Thirty-two of the vendors were cooking with either charcoal or LPG for 5 years or less, while 22 were cooking for more than 10 years. Fifty-seven of the cooking vendors currently work more than 5 days per week and 52 vendors worked more than 5 h per day.

Vendors with job duration longer than 10 years had a prevalence rate of 57.1% for cough compared to those who worked for 5 years or less who had a prevalence of 42.9% for cough (*P* = 0.007). Those with a job duration of 5 years or less had 91.7% prevalence rate for cold and chest illnesses compared to those with job duration of more than 10 years who had a 8.3% prevalence rate for cold and chest illnesses (*P* = 0.093) (Table 4).

**Table 4:** **Association between job duration and respiratory symptoms**.

Respiratory symptom	*n*	Job duration (years)				*P* value^a^
		Short		Long	
		1–5 frequency (%)	6–10 frequency (%)	11–15 frequency (%)	>15 frequency (%)	
Cough
No	64	35 (76.6)	14 (21.9)	3 (4.7)	12 (18.8)	0.007
Yes	21	6 (28.6)	3 (14.3)	4 (19.0)	8 (38.1)	
Phlegm
No	73	37 (50.7)	15 (20.5)	4 (5.5)	17 (23.3)	0.184
Yes	12	4 (33.3)	2 (16.7)	3 (25.0)	3 (25.0)	
Cold and chest illnesses
No	73	32 (43.8)	15 (20.5)	6 (8.2)	20 (27.4)	0.093
Yes	12	9 (75.0)	2 (16.7)	1 (8.3)	0 (0.0)	
Any respiratory symptom
No	45	25 (55.6)	8 (17.8)	3 (6.7)	9 (20.0)	0.353
Yes	40	16 (40.0)	9 (22.5)	4 (10.0)	11 (27.5)	

*^a^Fisher’s exact test (column 1–5 and 6–10 were combined as “short” and column 10–11 and >15 were combined as “long”)*.

### Association between respiratory problems and social factors in all vendors

Vendors who had pre-existing medical conditions such as asthma were more likely to have at least one respiratory symptom present (Crude OR = 4.31; 95% CI: 1.39; 13.39) (*P* = 0.034).

Those who currently smoke or have ever smoke were also more likely to experience respiratory symptoms (Crude OR = 1.81; 95% CI: 0.75, 4.35) compared to those who have never smoked. Vendors with pets (Crude OR = 1.17; 95% CI: 0.43, 3.18) and hobbies (OR = 2.97; 95% CI: 0.71, 12.38) were also more likely to have respiratory symptoms (Table 5).

**Table 5:** **Factors associated with respiratory symptoms**.

Variable	Any respiratory symptom present		Crude OR^a^ (95% CI)^a^	Adjusted OR^b^ (95% CI)^b^	*P* value^b^
	Yes	No	
Age (years)
≤34	25 (41.0)	36 (59.0)	2.40 (0.91; 6.34)	2.43 (0.82; 7.22)	0.110
>34	15 (62.5)	9 (37.5)	
Gender
Male	19 (47.5)	21 (52.5)	1.03 (0.44; 2.43)	2.22 (0.65; 7.55)	0.204
Female	21 (46.7)	24 (53.3)	
Cooking
Yes	34 (50.7)	33 (49.3)	2.06 (0.69; 6.13)	3.27 (0.92; 11.66)	0.068
No	6 (33.3)	12 (66.7)	
Smoking
Yes	19 (55.9)	15 (44.1)	1.81 (0.75; 4.35)	1.74 (0.51; 6.00)	0.380
No	21 (41.2)	30 (58.8)	
Pets
Yes	10 (50.0)	10 (50.0)	1.17 (0.43; 3.18)	1.62 (4.67; 5.65)	0.447
No	30 (46.2)	35 (53.8)	
Hobbies
Yes	7 (70.0)	3 (30.0)	2.97 (0.71; 12.38)	1.13 (0.18; 7.30)	0.895
No	33 (44.0)	42 (56.0)	
Pre-existing medical condition
Yes	8 (100.0)	0 (0.0)	4.31 (1.39; 13.39)	4.72 (1.12; 19.87)	0.034
No	32 (41.6)	45 (58.4)			

*^a^Simple logistic regression*.

*^b^Multiple logistic regression (Hosmer–Lemeshow goodness-of-fit test: *X* ^2^ = 11.44; df = 6; *P* = 0.076) (67.1% correctly classified by the model)*.

## Discussion

### General characteristics of vendors

A study on respiratory problems in this particular occupational group has not been previously documented in Brunei Darussalam. Nevertheless, a similar study (10) was conducted in Nigeria on male workers, focusing on the impact of wood smoke and oil fumes from a traditional barbecue cooking method. Smoking history were 33% (16/48) and 9% (3/32) for the case (*n* = 48) and control (*n* = 32) groups being current smokers (*P* = 0.014). Self-reported prevalences were also analyzed among the study participants.

The same study (10) showed that cough was the commonest symptom recorded; in both the test (59%) and control (27%) groups (OR = 3.1; 95% CI: 0.1, 5.8) with *P* value = 0.04. Results in this study also indicated that cough (50.7%) was the main respiratory symptom presented.

There was an increasing prevalence of respiratory symptoms (in particular, cough) among non-cooking vendors, vendors cooking with LPG, and vendors cooking with coal. The method of cooking may affect the prevalence of respiratory symptoms, as See et al. (5) demonstrated. The combustion of charcoal would produce more airborne impurities such as carbon monoxide (CO), volatile organic compounds (VOCs), and carbonyl compounds compared to the use of LPG.

There was an association between job duration of more than 10 years and respiratory symptoms of cough. Such respiratory tract symptoms in vendors exposed over the long-term can be explained by chronic irritation of airway due to exposure to irritants from cooking fumes. A previous study (11) suggested a dose–response pattern between length of exposure (mean exposure time = 16 years) to wood and charcoal smoke and chronic obstructive pulmonary disease.

Symptoms suggestive of lower respiratory tract involvement, such as cold and chest illnesses were higher among those with job duration of 5 years or less. This could be due to a lower prevalence among those who worked longer because of the survivor effect. An 11-year follow-up study (12) (1999) in China found evidence indicating the existence of a healthy-worker survivor effect associated with 11-year loss in FEV_1_ and dust exposure using a multivariate model. It is, however, contradictory to another study (13) in Port Harcourt, Nigeria, whereby findings had shown that outdoor cooking or roasting using charcoal <14 h daily for <10 years is not enough to cause respiratory problems.

### Strengths and limitations

The study had an acceptable response rate of 70.8%. We also utilized a translated Malay survey to ensure respondents’ comprehension.

However, no information was collected regarding whether the respondents had changed carriers before, i.e., from charcoal to LPG user or vice versa, which is a limitation of the study. The use of a self-administered questionnaire itself is subjective. Objective methods such as accurate air pollutant monitoring and lung function measures were not performed in this study.

## Conclusion

Food vendors in the open-air hawker center exposed to cooking fumes had more respiratory symptoms (50.7%) compared to merchandise sellers (33.3%). The prevalence of respiratory symptoms was associated with the type of fuel used for cooking (vendors cooking with charcoal were twice as likely to have cough compared to those cooking with LPG) and job duration (cooking vendors with a job duration more than 10 years were thrice more likely to have cough).

Based on these findings, control measures such as health education and smoking cessation programs, and regular monitoring of lung function of exposed vendors should be considered. The work environment may be examined regularly by measuring the level of airborne pollutants. Steps can be taken to improve ventilation and removal of the cooking fumes via exhaust ventilation. At the same time, vendors should consider the use of LPG rather than charcoal as the latter is associated with more respiratory symptoms.

We would also like to recommend carrying out further studies using a longitudinal design and to incorporate appropriate lung functions measurements such as spirometry to monitor any changes over time and also to observe whether there is any development of fixed airflow obstruction due to long-term occupational exposure to biomass or LPG as a source of fuel.

## Conflict of Interest Statement

The authors declare that the research was conducted in the absence of any commercial or financial relationships that could be construed as a potential conflict of interest.
